# 853. Leveraging a Collaboration Platform to Enhance Multidisciplinary Coordination and Patient Safety in Burn Care

**DOI:** 10.1093/jbcr/irag033.323

**Published:** 2026-04-06

**Authors:** Mack D Drake, Dallas Smith, Hali Wolf, Courtney Denton, Peter Yen

**Affiliations:** Burn and Reconstructive Centers of Virginia Chippenham, Richmond, VA; Joseph M Still Burn and Reconstructive Center/Burn and Reconstructive Centers of America, Augusta, GA; Burn and Reconstructive Centers of Virginia Chippenham, Richmond, VA; Burn and Reconstructive Center at Chippenham Hospital, Richmond, VA; Burn and Reconstructive Centers of Virginia Chippenham, Richmond, VA

## Abstract

**Introduction:**

Burn care demands highly coordinated input from surgery, critical care, nursing, wound care, rehabilitation, case management, and hospital administration. Traditional modes of communication—including email, paper notes, and EHR messages—often fail to support real-time, team-based coordination, leading to fragmented care and delayed interventions. As the demand for digitally integrated care delivery grows, the need for scalable, HIPAA-compliant communication platforms becomes increasingly urgent. We utilized an advanced collaboration platform for multidisciplinary communication in burn care, with goals to enhance real-time collaboration, improve the capture and tracking of consults and referrals as key performance indicators (KPIs), and advance patient safety and care quality.

**Methods:**

The platform was implemented across a national private physician group delivering burn, wound, hand, and reconstructive surgical care. Dedicated channels were created for provider updates, referrals, urgent consults, surgical planning, discharge coordination, and case management. Stakeholders—including physicians, APPs, nurses, rehab staff, and administrators—were granted role-based access. Metrics tracked over a 6-month pilot included platform utilization, consult and referral capture, and communication-related incident reports.

**Results:**

Shortly after implementation the platform's adoption increased dramatically, with activity rising from 449 interactions in the first month to 1439 in the sixth—a 320% increase. Captured referrals and consults rose by 52.9%, providing a significantly improved KPI database for operational reporting and strategic planning. Patient care indicators improved across multiple domains:

• MDRO-related events decreased by 60%.

• OT/PT consults were completed within 24 hours of admission nearly 100% of the time.

• Burn diagram documentation improved by 9%, reaching full compliance.

• Burn activation response time achieved 100% compliance within 30-minute threshold.

Communication response times between providers shortened, expediting surgical decisions, patient transfers, and discharge planning. Staff surveys reported enhanced satisfaction with clarity of roles, communication efficiency, and care coordination. Fewer adverse events linked to miscommunication were observed.

**Conclusions:**

Integrating an advanced collaboration platform into burn care workflows significantly improved multidisciplinary communication, enhanced real-time data capture for consults and referrals, and strengthened overall patient safety. This low-cost, secure, and scalable solution supports high-reliability operations and can be adapted across other high-acuity healthcare settings seeking to improve collaboration and KPI monitoring in pursuit of quality-driven care delivery.

**Applicability of Research to Practice:**

This platform enhances workflows in a busy national burn and reconstructive center network.

**Funding for the study:**

N/A.

